# A study on cognitive trajectory changes and predictive factors in middle-aged and older adults individuals with dual sensory impairment based on the health social determinants model

**DOI:** 10.3389/fpubh.2024.1489429

**Published:** 2024-12-17

**Authors:** Li Ma, Jiaxue Pang, Qiankun Liu, Pengyao Li, Juju Huang, Yang Xu, Hui Xie

**Affiliations:** College of Nursing, Bengbu Medical University, Bengbu, Anhui, China

**Keywords:** dual sensory impairment, cognitive trajectory, CHARLS, longitudinal study, middle-aged and older adults individuals

## Abstract

**Aim:**

This study aims to explore the cognitive trajectory changes in middle-aged and older adults individuals with dual sensory impairment (simultaneous visual and hearing impairment) and to identify the predictors of different trajectory changes.

**Methods:**

Based on the longitudinal data from the China Health and Retirement Longitudinal Study (CHARLS) from 2013 to 2020, data from 2,369 middle-aged and older adults individuals with dual sensory impairment were selected. A latent variable growth mixture model was constructed to analyze the cognitive function development trajectories in this population and to identify their predictive factors.

**Results:**

The cognitive function development trajectories in the middle-aged and older adults population can be categorized into three types: high cognitive level stable group, low cognitive level slowly declining group, and moderate cognitive level rapidly declining group. Logistic regression analysis showed that age (OR 30.544; 95% CI 9.35–99.754; *p* < 0.001), sleep duration (OR 0.559; 95% CI 0.343–0.909; *p* < 0.005), education (OR 0.009; 95% CI 0.003–0.025; *p* < 0.001), marital status (OR 2.122; 95% CI 1.457–3.090; *p* < 0.001), social participation (OR 0.499; 95% CI 0.379–0.658; *p* < 0.001), place of residence (OR 1.471; 95% CI 1.089–1.988; *p* < 0.001), and medical insurance (OR 0.353; 95% CI 0.169–0.736; *p* < 0.005) are predictive factors for cognitive function trajectories in this population.

**Conclusion:**

There is group heterogeneity in the cognitive function development trajectories among middle-aged and older adults individuals with dual sensory impairment. Factors such as less than 4 h of nighttime sleep, low social participation, alcohol consumption, and lack of medical insurance are modifiable risk factors for cognitive decline in this population. Preventive strategies should be formulated accordingly, especially for vulnerable groups, including older rural residents and those with lower educational attainment, to prevent cognitive function deterioration in middle-aged and older adults individuals with dual sensory impairment.

## 1 Introduction

The world’s population is rapidly aging, and the aging population in both developed and developing countries will continue to grow. It is estimated that by 2030, the global older adults population will reach 1.4 billion (1), With population aging, the prevalence of age-related vision impairment (VI), hearing impairment (HI), and dual sensory impairment (DSI; defined as concurrent VI and HI) is also increasing, becoming a global public health issue. The World Health Organization (WHO) reports that at least 2.2 billion people worldwide have vision impairment or blindness (2). It is estimated that by 2050, one in four people will have some degree of hearing loss, with age-related hearing loss being the most common (3). Studies have shown that sensory impairments are associated with limitations in daily activities (4), reduced social interactions (5), and decreased quality of life among older adults (6). They also increase the risk of cognitive decline and dementia (7).

Cognitive function is an important indicator of health status, directly affecting an individual’s ability to perform daily activities and overall quality of life. Older adults with cognitive decline are more likely to experience limited daily activities and require continuous care from family and social services, which increases the burden on family members and social insurance funds (8). Therefore, understanding cognitive decline and the factors that might mitigate it is crucial for early intervention and reducing dementia cases (9). There is a close relationship between sensory impairment and cognitive impairment, with vision and hearing impairments considered potentially modifiable risk factors for cognitive impairment in older adults (10). Research indicates that hearing impairment negatively affects communication and social participation in older adults, further increasing cognitive decline. Compared to individuals with a single sensory impairment (either hearing or vision impairment), older adults with dual sensory impairment are at a higher risk of cognitive decline (11).

Currently, research on cognitive decline in middle-aged and older adults individuals with dual sensory impairment is notably lacking. Older adults with sensory impairments face multiple physiological and social challenges, including loss of independence in daily life, marginalization in society, and the gradual depletion of life resources. Their cognitive decline is often the result of these cumulative resource losses (12). Most previous studies (13, 14) have focused on single sensory impairments, with limited research investigating the relationship between dual sensory impairment and cognitive trajectory changes in middle-aged and older adults populations. Furthermore, most studies (15, 16) have employed cross-sectional designs to examine the relationship between sensory impairment and cognitive risk, with a narrow focus on the factors influencing cognitive impairment caused by sensory impairment. This study adopts the “Social Determinants of Health” model, which encompasses multiple levels, ranging from individual to macro-social conditions (17). These include factors such as “health factors,” “individual lifestyles,” “social and community networks,” “structural social factors,” and “macro-social conditions.” This comprehensive framework provides direction and five specific dimensions for exploring the issue of cognitive decline in older adults with sensory impairments. Therefore, this study, supported by the “Social Determinants of Health” model, will investigate the factors affecting cognitive function in middle-aged and older adults individuals with dual sensory impairment. Based on these findings, the study aims to propose multidimensional strategies to improve cognitive function in this population.

This study aims to explore the following three questions: (1) What are the cognitive trajectories of middle-aged and older adults individuals with dual sensory impairment? (2) What are the predictive factors influencing cognitive levels in middle-aged and older adults individuals with dual sensory impairment? (3) What intervention measures should be developed in the future based on these factors?

## 2 Materials and methods

### 2.1 Data collection

**Figure 1:**
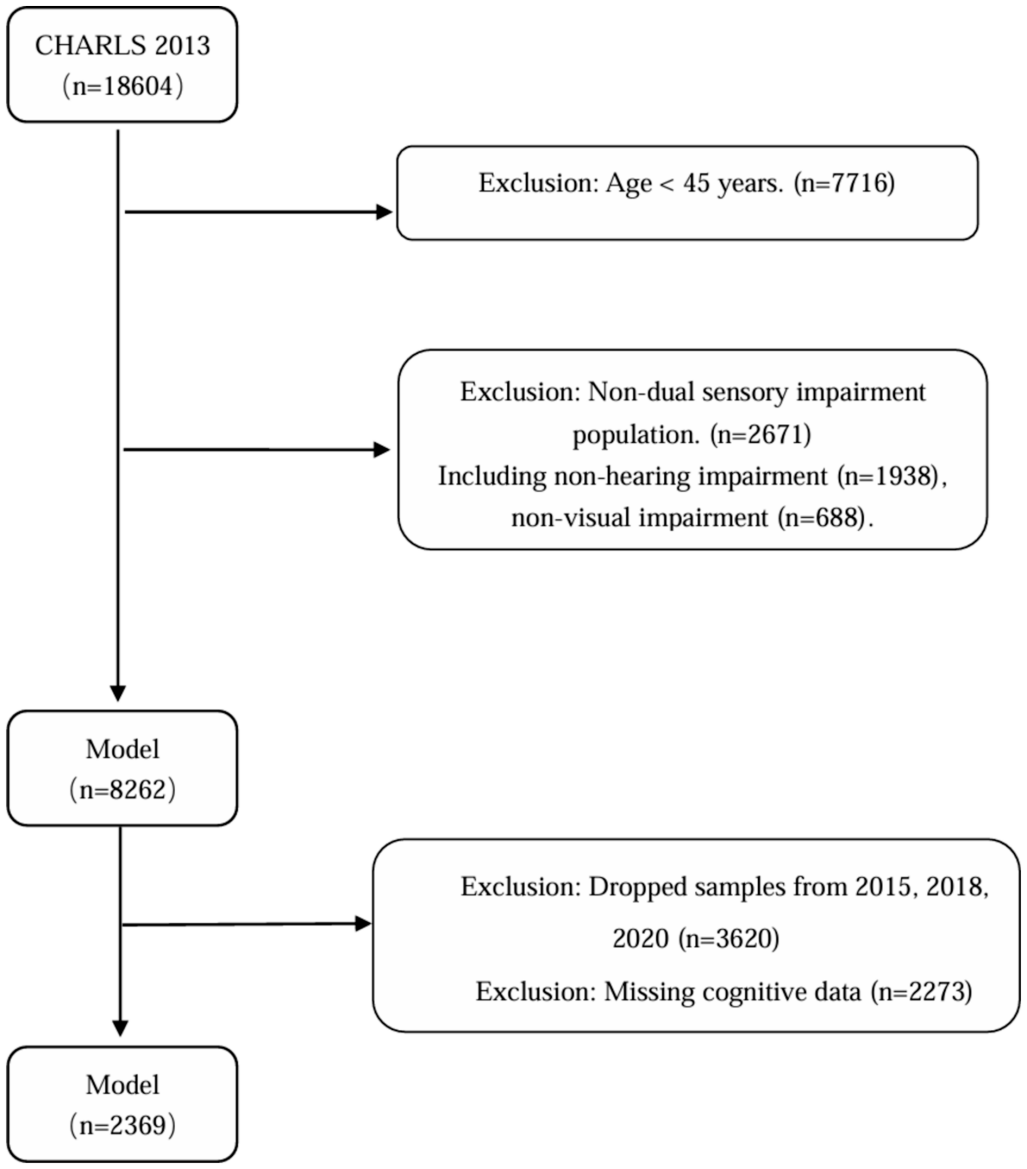
Flowchart of the CHARLS population selection process.

The China Health and Retirement Longitudinal Study (CHARLS) is a large-scale, interdisciplinary longitudinal study initiated by the National School of Development (NSD) at Peking University. CHARLS uses a stratified, multistage probability-proportional-to-size random sampling strategy to survey middle-aged and older adults individuals aged 45 and above across 28 provinces (autonomous regions and municipalities) in China, assessing the social, economic, and health status of community residents. The national baseline survey of the study was conducted in 2011, followed by follow-up surveys every 2–3 years in 2013, 2015, 2018, and 2020 (18). This study utilized the longitudinal follow-up data from CHARLS between 2013 and 2020. According to the study objectives, the inclusion criteria were: (1) age ≥45 years in the 2013 baseline survey; (2) presence of both vision and hearing impairments at baseline; and (3) participation in at least three follow-up surveys. The exclusion criteria were: (1) individuals with missing health status or basic information, (2) individuals with missing vision, hearing, or cognitive information at baseline, and (3) individuals with missing cognitive data during follow-up, (4) Patients with severe hearing and vision impairment (deafness and blindness) who cannot complete a cognitive function assessment. Finally, this study included a total of 2,369 middle-aged and older adults individuals aged 45 and above with dual sensory impairment. The specific screening flowchart is shown below (Figure 1).

All participants provided informed consent, and the protocol was approved by the Peking University Institutional Review Board (Approval No: IRB00001052-11015). All procedures performed in studies involving human participants were in accordance with the ethical standards of the institutional and/or national research committee and with the 1964 Helsinki Declaration and its later amendments or comparable ethical standards.

### 2.2 Variables and measurement methods

#### 2.2.1 Cognitive assessment

The Mini-Mental State Examination (MMSE) is widely used worldwide to assess cognitive function (19). The scale was specifically developed and published by Folstein et al. in 1975 as a tool for quickly screening cognitive impairment within a short period (19). This scale is widely used in China, and previous domestic studies suggest that the scale’s Cronbach’s alpha coefficient is 0.833, and the test–retest reliability is 0.924 (20). In line with previous research, cognitive function was measured in two domains: episodic memory and global cognition, with a total score range of 0–31, where higher scores indicate better cognitive function (21). Episodic memory was assessed using immediate and delayed word recall tests. After the interviewer randomly read 10 Chinese words, participants were asked to recall them immediately and again after a few minutes. The episodic memory score was the sum of recalled items, ranging from 0 to 20. Global cognition was assessed through three tasks: orientation (day, month, date, season, and year), calculation (serial subtraction of 7 from 100 five times), and visuospatial ability (drawing overlapping pentagons). Participants received one point for each correct item, with scores ranging from 0 to 11. The China Health and Retirement Longitudinal Study survey personnel received professional training on the MMSE for each wave to minimize systematic differences as much as possible.

#### 2.2.2 Assessment of dual sensory impairment

Participants who simultaneously reported vision and hearing impairments were considered to have dual sensory impairment. The self-reported data on vision impairment consisted of two questions: (1) “How good is your eyesight for seeing things at a distance (with glasses or corrective lenses), such as recognizing a friend across the street?” and (2) “How good is your eyesight for seeing up close (with glasses or corrective lenses), such as reading ordinary newspaper print?” For each question, responses included “excellent,” “very good,” “good,” “fair,” or “poor.” If respondents reported their vision as “fair” or “poor” (either for distance or near vision), they were classified as having vision impairment in this study. Hearing impairment was assessed with one question: “Is your hearing excellent, very good, good, fair, or poor?” If participants reported their hearing as “fair” or “poor,” they were classified as having hearing impairment.

#### 2.2.3 Covariates

Based on the health social determinants model, this study uses binary coding (22) and the actual conditions of CHARLS data, this study selected five levels of associated factors, including: (1) Health Factors: Gender (1 = male, 2 = female); Age (1 = 45–59 years, 2 = 60–79 years, 3 = 80 years and above); Chronic diseases (1 = none, 2 = one chronic disease, 3 = two or more chronic diseases); Self-rated health, measured by the question: “How would you rate your current health status?” The answers include “very good, good, fair, poor, very poor.” In this study, “very good” and “good” are classified as good, “fair” as average, and “poor” and “very poor” as poor (1 = good, 2 = average, 3 = poor); Disability status (1 = no, 2 = yes). (2) Individual Lifestyle: Alcohol consumption (1 = yes, 2 = no); Sleep duration (1 = less than 4 h per night, 2 = 4–6 h per night, 3 = more than 6 h per night). (3) Social and Community Networks: Marital status (1 = married, 2 = not married); Social participation was measured using question DA056, which asked respondents whether they had engaged in any of the following 12 social activities in the past month: (a) Interacting with friends; (b) Playing mahjong, chess, cards, or going to a community club; (c) Helping family members, friends, or neighbors who do not live with them; (d) Participating in sports clubs, social clubs, or other clubs; (e) Joining community organizations; (f) Engaging in volunteer or charity work; (g) Taking care of sick or disabled adults who do not live with them; (h) Attending educational or training courses; (i) Investing in stocks; (j) Using the internet; (k) Participating in other social activities; (l) None of the above. If respondents selected any of the first 11 options, they were classified as participating in social activities; otherwise, they were classified as not participating (23) (1 = yes, 2 = no). (4) Social Structural Factors: Years of education (1 = 0 years, 2 = 1–6 years, 3 = 7–12 years, 4 = 13 years and above); Retirement status (1 = not retired, 2 = retired). (5) Macro Social Conditions: Residence (1 = urban, 2 = rural); Health insurance (1 = no, 2 = yes).

## 3 Statistical analysis

Statistical analyses were conducted using SPSS version 28.0 for descriptive statistics, χ^2^ tests, and unordered multinomial logistic regression analyses, with a significance level of *α* = 0.05. Latent profile analysis of cognitive scores from 2013 to 2020 was performed using Mplus version 8.3. The fit indices included the Akaike Information Criterion (AIC), the Bayesian Information Criterion (BIC), and the sample-size adjusted BIC (aBIC), with smaller values indicating better fit. An entropy value ≥0.8 indicates that the classification accuracy exceeds 90%, with values closer to 1 reflecting higher accuracy. Model fit differences were assessed using the Likelihood Ratio Test (LMR) and the Bootstrap-based Likelihood Ratio Test (BLRT). A *p*-value <0.05 indicates that the new model is superior to the previous one. The best model was selected based on a comprehensive comparison of the fit indices for each model.

## 4 Results

### 4.1 Descriptive characteristics

A total of 2,369 participants were included in the baseline survey of this study, with an average age of 62.35 ± 9.78 years (ranging from 45 to 92 years). Among them, 1,019 (43.0%) were male, and 1,350 (57.0%) were female. Participants from rural areas accounted for 1,570 (66.3%), while those from urban areas totaled 799 (33.7%). A total of 1,918 (81.0%) were married, and 451 (19.0%) were not married. Regarding education level, 1,763 (74.4%) had six or fewer years of schooling, 536 (22.6%) had 7–12 years of education, and 70 (3.0%) had 13 or more years of education. Detailed baseline characteristics of the participants are shown in Table 1.

**Table 1:** Comparison of basic characteristics across latent classes of cognitive levels in older adults with dual sensory impairment (*n* = 2,369).

Variable	C1 (*n* = 804)	C2 (*n* = 708)	C3 (*n* = 857)	*χ^2^/F*	*p*
**Age (years)**				359.703	<0.001
45–59	493 (61.3)	302 (42.7)	175 (20.4)		
60–79	307 (38.2)	390 (55.1)	579 (67.6)		
≥80	4 (0.5)	16 (0.2)	103 (12)		
**Gender, *n* (%)**				69.104	<0.001
Male	364 (45.3)	362 (51.1)	293 (34.2)		
Female	440 (54.7)	346 (48.9)	564 (65.8)		
**Chronic diseases, *n* (%)**				10.364	<0.035
None	326 (40.5)	259 (36.6)	293 (34.2)		
One	192 (23.9)	157 (22.2)	222 (25.9)		
Two or more	286 (35.6)	292 (41.2)	342 (39.9)		
**Self-rated health, *n* (%)**				91.700	<0.001
Good	109 (13.6)	77 (10.9)	102 (11.9)		
Average	532 (66.2)	409 (57.8)	398 (46.4)		
Poor	163 (20.2)	222 (31.3)	357 (41.7)		
**Disability status, *n* (%)**				96.461	<0.001
No	689 (85.7)	541 (76.4)	557 (65.0)		
Yes	115 (14.3)	167 (23.6)	300 (35.0)		
**Alcohol consumption, *n* (%)**				80.724	<0.001
Yes	444 (55.2)	384 (54.2)	305 (35.6)		
No	360 (44.8)	324 (45.8)	552 (64.4)		
**Sleep duration, *n* (%)**				120.740	<0.001
<4 h	43 (0.5)	70 (10.0)	190 (22.2)		
4–6 h	446 (55.5)	339 (47.9)	345 (40.3)		
>6 h	315 (39.1)	299 (42.1)	322 (37.5)		
**Marital status, *n* (%)**				108.574	<0.001
Married	722 (89.9)	594 (83.9)	602 (70.2)		
Unmarried	82 (10.1)	114 (16.1)	255 (29.8)		
**Social participation, *n* (%)**				96.381	<0.001
Yes	536 (66.7)	393 (55.5)	366 (42.7)		
No	268 (33.3)	315 (44.5)	491 (57.3)		
**Years of education, *n* (%)**				998.598	<0.001
0 year	35 (4.4)	187 (26.4)	571 (66.7)		
1–6 years	328 (40.8)	391 (55.2)	251 (29.3)		
7–12 years	388 (48.3)	118 (16.7)	30 (3.5)		
13 years and above	53 (6.5)	12 (1.7)	5 (0.5)		
**Retirement status, *n* (%)**				78.808	<0.001
Not retired	589 (73.3)	480 (67.8)	455 (53.1)		
Retired	215 (26.7)	228 (32.2)	402 (46.9)		
**Residence, *n* (%)**				61.119	<0.001
Urban	356 (44.3)	207 (29.3)	236 (27.5)		
Rural	448 (55.7)	501 (70.7)	621 (72.5)		
**Health insurance, *n* (%)**				47.048	<0.001
No	18 (2.2)	16 (2.3)	71 (82.3)		
Yes	786 (97.8)	692 (97.7)	786 (91.7)		

### 4.2 Cognitive trajectory results for older adults with dual sensory impairment

Setting up models with 1–4 categories. The results showed that as the number of categories increased, the values for AIC, BIC, and aBIC decreased, while the Entropy value also changed. Although AIC, BIC, and aBIC values generally decreased in this study, a significant inflection point was observed in the three-category model, where the rate of decrease markedly slowed. This indicates that the four-category model did not show significant improvement over the three-category model in terms of AIC, BIC, and aBIC indices. Additionally, the four-category model is merely an extension of the three-category model and did not provide new theoretical contributions. When profiles have similar theoretical meanings, a simpler profile model should be selected. Therefore, based on the fit indices and the practical significance of the latent categories, the three-category model was ultimately chosen as the best model. See Table 2.

**Table 2:** Results of the latent class growth mixture model (LGMM) for cognitive levels.

Model	*k*	*AIC*	*BIC*	*aBIC*	*Entropy*	*LMR*	*BLRT*	Class probability
1	6	63,257.112	63,291.734	63,272.670	–	–	–	–
2	9	57,193.573	57,245.505	57,216.910	0.925	<0.001	<0.001	0.456/0.544
3	12	55,517.453	55,586.696	55,548.569	0.883	<0.001	<0.001	0.338/0.363/0.299
4	15	54,733.252	54,819.805	54,772.147	0.863	<0.001	<0.001	0.158/0.315/0.256/0.271

K, number of freely estimated parameters; AIC, Akaike Information Criterion; BIC, Bayesian Information Criterion; aBIC, sample-size adjusted Bayesian Information Criterion; Entropy, information entropy; LMR, Lo–Mendell–Rubin likelihood ratio test; BLRT, Bootstrap likelihood ratio test.

### 4.3 Cognitive trajectory groupings for older adults with dual sensory impairment

**Figure 2:**
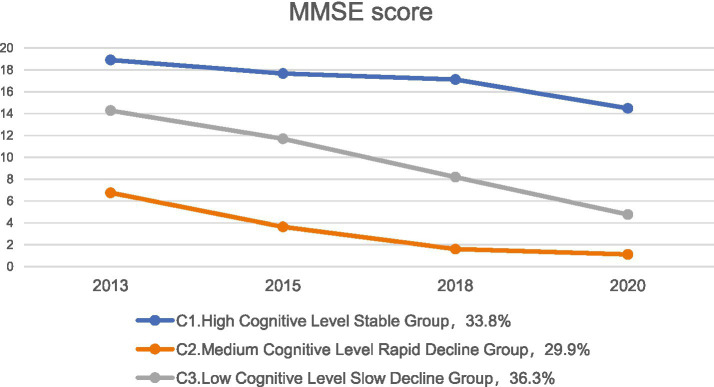
Cognitive trajectory of older adults with dual sensory impairment.

The LGMM model with three categories estimated the following results: Group C1: This group had the highest average cognitive score (*α* = 19.067, *p* < 0.001) and exhibited a stable trend (*β* = −1.296, *p* < 0.001). It was named the High Cognitive Level Stable Group, comprising 33.8% of the sample. Group C3: This group had a higher baseline average cognitive score (*α* = 14.593, *p* < 0.001) but showed a rapid decline (*β* = −3.184, *p* < 0.001). It was named the Medium Cognitive Level Rapid Decline Group, comprising 36.3% of the sample. Group C2: This group had a lower average cognitive score (*α* = 6.126, *p* < 0.001) and a faster decline (*β* = −1.977, *p* < 0.001). It was named the Low Cognitive Level Slow Decline Group, comprising 29.9% of the sample. The cognitive level development trajectories are shown in Figure 2. The estimated values and test results for the intercepts and slopes of each latent cognitive level category are presented in Table 3.

**Table 3:** Intercept and slope estimates and test results for each latent class of cognitive levels in older adults with dual sensory impairment.

Category	*n*	Intercept				Slope			
		Estimated value	SE	*t*	*p*	Estimated value	SE	*t*	*p*
C1 High Cognitive Level Stable Group	804	19.067	0.154	123.902	<0.001	−1.296	0.114	−11.332	<0.001
C2 Low Cognitive Level Slow Decline Group	857	6.126	0.224	27.393	<0.001	−1.977	0.068	−29.003	<0.001
C3 Medium Cognitive Level Rapid Decline Group	708	14.593	0.395	36.939	<0.001	−3.184	0.110	−28.963	<0.001

### 4.4 Univariate analysis of cognitive level categories in older adults with dual sensory impairment

The results of the univariate analysis showed significant differences between the categories in terms of age, gender, marital status, years of education, alcohol consumption, sleep duration, disability status, retirement status, depression, social participation, number of chronic diseases, self-rated health, and health insurance. Compared to the High Cognitive Level Stable Group, the Low Cognitive Level Slow Decline Group and the Medium Cognitive Level Rapid Decline Group had a higher proportion of individuals aged 60 and above (38.68% vs. 57.34% vs. 79.57%). Among the three groups, the Medium Cognitive Level Rapid Decline Group had the highest proportion of females (65.81% vs. 58.20% vs. 48.88%). Rural residents were more prevalent in all three groups, with the highest proportions in the Low Cognitive Level Slow Decline Group (70.76%) and the Medium Cognitive Level Rapid Decline Group (72.46%). There were also differences in years of education among the groups. The High Cognitive Level Stable Group had a higher proportion of individuals with 6 or more years of education compared to the Low Cognitive Level Slow Decline Group and the Medium Cognitive Level Rapid Decline Group (54.85% vs. 18.36% vs. 4.08%). In the Medium Cognitive Level Rapid Decline Group, a large proportion had 6 or fewer years of education (95.92%). Compared to the High Cognitive Level Stable Group, individuals in the Low Cognitive Level Slow Decline Group and the Medium Cognitive Level Rapid Decline Group experienced more severe disability, shorter sleep duration, and less social participation.

### 4.5 Multivariate analysis of cognitive level categories in older adults with dual sensory impairment

In this study, the three latent cognitive level trajectory categories were used as dependent variables, with the “Low Cognitive Level Slow Decline Group” serving as the reference group, in a multivariate unordered logistic regression analysis. The multivariate analysis results showed: High Cognitive Level Stable Group: Compared to the Low Cognitive Level Slow Decline Group, individuals in the High Cognitive Level Stable Group were more likely to report good or average self-rated health (OR = 1.909; 95% CI: 1.373–2.653; *p* < 0.001), no disability (OR = 1.613; 95% CI: 1.146–2.271; *p* < 0.006), being married (OR = 2.122; 95% CI: 1.457–3.090; *p* < 0.001), and high social participation (OR = 0.499; 95% CI: 0.379–0.658; *p* < 0.001). Medium Cognitive Level Rapid Decline Group: Compared to the Low Cognitive Level Slow Decline Group, individuals in the Medium Cognitive Level Rapid Decline Group were more likely to have low education years (OR = 0.090; 95% CI: 0.063–0.570; *p* < 0.003), consume alcohol (OR = 1.965; 95% CI: 1.558–2.479; *p* < 0.001), lack health insurance (OR = 0.310; 95% CI: 0.168–0.571; *p* < 0.001), and have short sleep duration (OR = 0.602; 95% CI: 1.558–2.479; *p* < 0.003). See Table 4 for details.

**Table 4:** Logistic regression analysis of factors affecting cognitive level changes in older adults with dual sensory impairment.

Dependent variable	Independent variable	*β*	SE	*Waldχ^2^*	*p*	*OR*	95%CI
C1 vs. C2	Intercept	−2.404	0.810	8.803	0.003	-	-
	**Age (Reference group: ≥80)**						
	45–59	3.419	0.604	32.059	<0.001	30.544	9.35–99.754
	60–79	2.379	0.593	16.113	<0.001	10.794	3.37–34.489
	**Gender (Reference group: Female)**						
	Male	−0.200	0.151	1.760	0.185	0.819	0.610–1.100
	**Chronic diseases (Reference group: two or more)**						
	No	−0.356	0.167	4.535	0.033	0.701	0.505–0.972
	One	−0.231	0.180	1.641	0.200	0.794	0.558–1.130
	**Self-rated health (Reference group: poor)**						
	Good	0.596	0.253	5.520	0.019	1.814	1.104–2.981
	Average	0.646	0.168	14.803	<0.001	1.909	1.373–2.653
	**Disability status (Reference group: yes)**						
	No	0.478	0.174	7.527	0.006	1.613	1.146–2.271
	**Alcohol consumption (Reference group: no)**						
	Yes	0.651	0.138	22.344	<0.001	1.917	1.464–2.511
	**Sleep duration (Reference group: >6 h)**						
	<4 h	−0.582	0.248	5.500	0.019	0.559	0.343–0.909
	4–6 h	0.273	0.148	3.414	0.065	1.314	0.984–1.754
	**Marital status (Reference group: unmarried)**						
	Married	0.752	0.192	15.380	<0.001	2.122	1.457–3.090
	**Social participation (Reference group: no)**						
	Yes	−0.694	0.141	24.416	<0.001	0.499	0.379–0.658
	**Years of education (Reference group: 13 years and above)**						
	0 year	−4.748	0.538	77.806	<0.001	0.009	0.003–0.025
	1–6 years	−1.893	0.507	13.932	<0.001	0.151	0.056–0.407
	7–12 years	−0.006	0.534	0.000	0.991	0.994	0.349–2.832
	**Retirement status (Reference group: retired)**						
	Not retired	0.592	0.157	14.177	<0.001	1.808	1.328–2.460
	**Residence (Reference group: rural)**						
	Urban	0.386	0.154	6.325	<0.001	1.471	1.089–1.988
	**Health insurance (Reference group: yes)**						
	No	−1.042	0.375	7.715	0.005	0.353	0.169–0.736
C3 vs. C2	Intercept	−1.478	0.670	4.872	0.027	-	-
	**Age (Reference group: ≥80)**						
	45–59	1.925	0.326	34.848	<0.001	6.858	3.619–12.997
	60–79	1.213	0.308	15.532	<0.001	3.365	1.840–6.152
	**Gender (Reference group: Female)**						
	Male	0.177	0.132	1.811	0.178	1.194	0.922–1.547
	**Chronic diseases (Reference group: two or more)**						
	No	−0.260	0.144	3.277	0.070	0.771	0.582–1.022
	One	−0.293	0.154	3.645	0.056	0.746	0.552–1.008
	**Self-rated health (Reference group: poor)**						
	Good	0.109	0.217	0.253	0.615	1.115	0.729–1.706
	Average	0.289	0.138	4.375	0.036	1.335	1.018–1.750
	**Disability status (Reference group: yes)**						
	No	0.233	0.139	2.795	0.095	1.275	0.961–1.660
	**Alcohol consumption (Reference group: no)**						
	Yes	0.676	0.119	32.477	<0.001	1.965	1.558–2.479
	**Sleep duration (Reference group: >6 h)**						
	<4 h	−0.508	0.188	7.271	0.007	0.602	1.558–2.479
	4–6 h	0.083	0.129	0.408	0.523	1.086	0.843–1.399
	**Marital status (Reference group: unmarried)**						
	Married	0.425	0.151	7.871	0.005	1.529	1.137–2.057
	**Social participation (Reference group: no)**						
	Yes	−0.386	0.120	10.336	0.001	0.680	0.537–0.860
	**Years of education (Reference group: 13 years and above)**						
	0 year	−1.663	0.562	8.768	0.003	0.190	0.063–0.570
	1–6 years	−0.313	0.555	0.319	0.572	0.731	0.246–2.169
	7–12 years	0.352	0.585	0.361	0.548	1.422	0.451–4.478
	**Retirement status (Reference group: retired)**						
	Not retired	0.243	0.130	3.506	0.061	1.275	0.989–1.644
	**Residence (Reference group: rural)**						
	Urban	−0.004	0.138	0.001	0.976	0.996	0.761–1.304
	**Health insurance (Reference group: yes)**						
	No	−1.172	0.312	14.072	<0.001	0.310	0.168–0.571

## 5 Discussion

This study analyzed the cognitive function trajectories over 8 years among middle-aged and older adults individuals aged 45 years and above with dual sensory impairment in China, using nationally representative CHARLS survey data. The findings revealed that most individuals with dual sensory impairment exhibited a decline in cognitive trajectories, with approximately one-quarter already showing signs of mild cognitive impairment at baseline. Therefore, analyzing the cognitive function trajectory changes and associated predictive factors in older adults with sensory impairments is of great significance for preventing and improving cognitive impairment in this population.

### 5.1 Cognitive function heterogeneity among older adults with dual sensory impairment

Regarding cognitive trajectories, there is heterogeneity in the cognitive trajectories of different middle-aged and older adults individuals. A study on cognitive function trajectories in individuals aged 55 years and older (24) identified three distinct trajectories: “persistently low cognitive function” (22.2%), “persistently moderate cognitive function” (37.9%), and “persistently high cognitive function” (39.9%). Additionally, other studies (25) classified cognitive trajectories into stable average, high and stable, and declining trends, with the overall decline trend being less than 10%. Notably, our study found that 36.3% of middle-aged and older adults individuals with dual sensory impairment belonged to the group with rapidly declining cognitive trajectories, significantly higher than the proportions reported in the aforementioned studies. This aligns with findings from a European study on older adults (26), indicating that cognitive decline in individuals with dual sensory impairment is more pronounced than in those with either visual or hearing impairment or no sensory impairment. Hypotheses have been proposed to explain the association between sensory and cognitive functions. According to the sensory deprivation hypothesis suggests (27) that prolonged reduction in sensory input leads to neuronal atrophy, resulting in cognitive decline in this population.

Our study demonstrates that middle-aged and older adults individuals aged 45 years and above in China with dual sensory impairment also exhibit multiple cognitive function development trajectories. This supports the hypothesis that cognitive function development among older adults with sensory impairments is a heterogeneous process rather than a homogeneous average process. In our study, the degree of cognitive decline varied among different subgroups. Specifically, individuals who were middle-aged (45–59 years), free from chronic diseases, highly educated (education years >12), had high social participation, and were free from disability were more likely to maintain good and stable cognitive function levels. Conversely, those who were older (60 years and above), had multiple chronic diseases (≥2), were less educated (education years <6), had low social participation, slept less than 4 h at night, consumed alcohol regularly, and mostly lived in rural areas were more likely to experience rapid cognitive decline. Therefore, communities should include vulnerable groups at risk of cognitive decline in high-risk populations for regular cognitive impairment screening, with particular attention to special populations such as those with visual and hearing impairments.

### 5.2 Predictive factors for cognitive function trajectories in older adults with dual sensory impairment

#### 5.2.1 Health factors

Using the low cognition and slow decline group as the reference group, this study found significant differences in predictive factors between the stable high cognition group and the rapid middle cognition decline group. In the stable high cognition group, self-rated health, the number of chronic diseases, pain, and disability were statistically significant. This may be because, in the rapid middle cognition decline group, cognitive function might have already been significantly impaired, with the disease progressing to a stage where other health factors (such as brain structural changes or neurodegenerative diseases) primarily drive the cognitive decline process. At this stage, factors like chronic disease, pain, or disability have relatively less impact and are insufficient to significantly alter the trajectory of cognitive function.

This study revealed that sensory-impaired older adults aged >60 years and those with disabilities are more likely to experience cognitive deterioration, consistent with previous research findings (28). As with many chronic diseases, aging is the most significant factor influencing the development of Alzheimer’s disease and related dementias (29). Even among older adults without lifelong dementia, cognitive decline and neurodegenerative changes become evident with age, driven by shared pathophysiological mechanisms such as abnormal autophagy, mitochondrial dysfunction, cellular senescence, epigenetic changes, cerebrovascular dysfunction, inflammation, and lipid dysregulation (30). Furthermore, disabled older adults often experience limited physical functionality, preventing them from engaging in normal daily activities and exercise. This lack of activity leads to insufficient brain stimulation, particularly in areas critical for cognitive functions such as memory, attention, and executive function (31). Prolonged physical inactivity is also associated with reduced brain plasticity, thereby accelerating cognitive decline (32).

#### 5.2.2 Individual lifestyle

At the individual lifestyle level, this study found that alcohol consumption and sleeping less than 4 h per night had an impact on cognition. Compared to non-drinkers, those who consumed alcohol were more likely to experience cognitive decline, consistent with a longitudinal study in the United States on older adults with dual sensory impairment (33). This study found that long-term alcohol consumption is associated with an increased risk of Alzheimer’s disease and other forms of dementia. Excessive drinking, in particular, can have direct toxic effects on the brain, leading to neuronal damage, reduced brain volume, and especially hippocampal atrophy, a region closely related to memory and cognitive function (34). Additionally, alcohol can induce neuroinflammation, oxidative stress, and apoptosis, further accelerating cognitive decline (35). Avoiding alcohol consumption can mitigate these risks, thus serving as a protective factor for maintaining cognitive stability. However, a recent meta-analysis (36) suggested a U-shaped association between alcohol consumption and dementia risk, with the lowest risk observed at four drinks per week, while consuming 23 or more drinks per week was associated with higher dementia risk. Another dose–response meta-analysis (37) identified a nonlinear (J-shaped) association between alcohol intake and the risk of cognitive impairment and dementia. These findings highlight ongoing debates about the dose–response relationship between alcohol consumption and cognitive function. Further research is needed to explore the relationship between alcohol consumption and six specific domains of cognitive impairment. In providing alcohol consumption guidance for older adults with dual sensory impairment, it is important to assess drinking behaviors and cognitive dimensions comprehensively and, based on individual circumstances, encourage either abstinence or moderate drinking.

This study also found that individuals sleeping less than 4 h per night were more likely to fall into adverse cognitive trajectories (low baseline cognition with deterioration). A quantitative meta-analysis showed that both insufficient sleep duration (<4 h per night or total daily sleep) and excessive sleep (>10 h per night or >12.5 h total daily sleep) increase the risk of all-cause cognitive impairment and Alzheimer’s dementia (AD) (38), consistent with our findings. However, another study suggested a general J-shaped relationship between objective sleep duration and cognitive performance, where the association between short objective sleep duration (<6 h) and poor cognitive function was not statistically significant (39). This inconsistency may be due to sleep duration data often relying on self-reports, as well as differences in study populations from various regions, countries, and ethnic groups, leading to variability in sleep characteristics (e.g., time in bed, sleep duration, and daytime sleepiness). The potential mechanism by which short sleep duration leads to cognitive decline is associated with higher incidence rates of gray matter atrophy in the frontal and temporal lobes among older adults, which may impair memory in individuals with dual sensory impairment (40). A large longitudinal study (41) investigated the prospective impact of changes in sleep duration on sensory impairments in individuals aged ≥65 years. It found that individuals with persistently short or long sleep durations were at higher risk of sensory impairment compared to those with normal sleep durations. These findings confirm the close and bidirectional relationship between sleep duration, sensory impairment, and cognitive function. Therefore, attention should be paid to the sleep patterns of older adults with dual sensory impairment. Promoting physical exercise is a simple strategy to address sleep problems in older adults, as it facilitates relaxation and increases core body temperature, aiding in the initiation and maintenance of sleep (42).

#### 5.2.3 Social and community networks

In the realm of social and community networks, older adults with dual sensory impairments who are unmarried (including widowed and divorced) and lack social activities are more likely to experience cognitive decline. Compared to married individuals, unmarried older adults are more prone to cognitive deterioration, which is consistent with findings from a study in the United States (43), this study found that married individuals performed better in memory assessments and had a lower risk of dementia compared to cohabiting, single, divorced, and widowed individuals. Existing research (44) attributes cognitive decline related to marital status to two main causal models: the resource model and the stress model. The resource model posits that the loss of marriage results in a loss of financial, social, practical, and emotional resources, which increases the risk of cognitive decline. The stress model suggests that losing a partner introduces stress, such as grief and adapting to new daily routines, which also heightens the risk of cognitive decline. However, some studies (43, 45) indicate that the type of marital loss affects cognitive function differently, with some individuals experiencing improved cognition while others deteriorate. Divorced individuals tend to perform better, while widowed or separated but not divorced individuals show worse outcomes. This study, constrained by database limitations, only examined the effects of marital status on cognition in those with sensory impairments, without considering other types of marital loss. Future research could provide a more detailed and comprehensive analysis of how different types of marital loss affect cognitive function.

Social engagement, as one of the three pillars of successful aging, is a critical pathway to achieving positive aging (46). Previous research has often overlooked social participation among individuals with sensory impairments. This study found that good social engagement is associated with a greater likelihood of experiencing positive cognitive trajectories in older adults. Individuals typically rely on both visual and auditory senses to perform tasks in daily life and social activities. For those with dual sensory impairments, declines in auditory and visual functions restrict their social interactions, and the long recovery process for sensory impairments can lead to feelings of loneliness and frustration. This often results in reduced outdoor social activities, and prolonged social isolation contributes to cognitive decline (47). Related research suggests that experiences of loneliness can alter brain activity in areas associated with vision, attention, and emotional processes (48, 49), another explanation is that insufficient social interaction may affect neurogenesis and synaptic density, reducing the brain’s ability to compensate for neurodegenerative damage related to Alzheimer’s disease (48). These findings underscore the importance of incorporating social engagement as an intervention before or during the preclinical stage of dementia. Therefore, communities and families can enhance indoor engagement by improving accessibility, leveraging urban and rural advantages to create social exchange platforms for individuals with sensory impairments, exploring various forms of social activities, and actively encouraging participation to improve social engagement and potentially mitigate or delay cognitive decline.

#### 5.2.4 Structural social factors

This study found that years of education and retirement status influence cognitive trajectory development. Higher education levels help slow cognitive decline in older adults. Current evidence suggests (50) that education enhances cognitive abilities by building a broad foundation of specific knowledge and skills, which, in turn, influence various cognitive abilities across development. Studies have shown (51) that compared to individuals with lower educational attainment, highly educated older adults exhibit greater cortical thickness in brain regions such as the cingulate cortex, transverse temporal cortex, insula, and isthmus. This supports greater brain reserve, which allows individuals with high cognitive reserve to prevent or delay β-amyloid deposition (52), mitigating the impact of age-related brain changes on cognitive function. Additionally, older adults with sensory impairments and higher education levels may utilize cognitive resources to compensate for sensory loss, thereby delaying cognitive decline (53). However, educational attainment is a relatively immutable risk factor. In the context of the digital age, families and communities can teach middle-aged and older adults individuals to use digital devices, such as smartphones, and leverage multimedia tools to help them access more knowledge.

This study also found that retired older adults exhibited lower cognitive levels. This may be attributed to sensory-impaired older adults individuals experiencing a dual loss of social status and bodily autonomy shortly after retirement, leading to heightened feelings of social isolation and deprivation of physical self-control. These factors negatively impact their mental health, thereby affecting cognitive levels (54). During employment, work often requires continuous cognitive activities such as thinking, decision-making, and problem-solving, which help keep the brain active. After retirement, the reduction in cognitive stimulation may lead to decreased brain activity, triggering cognitive decline (55). To prevent this, it is recommended that older adults actively engage in social activities, seek new cognitive challenges, and pursue learning opportunities post-retirement. Maintaining a positive psychological state can help delay cognitive decline.

#### 5.2.5 Macro-social conditions

The regression analysis of macro-social conditions affecting the cognitive levels of older adults with dual sensory impairment revealed that both residential location and type of medical insurance played protective roles. Older adults with dual sensory impairment living in urban areas and participating in urban employee medical insurance demonstrated relatively better cognitive levels. This may be attributed to the prioritization of urban areas by the national and local governments in China, leading to better economic development, public health infrastructure, and policy implementation compared to non-urban areas. Studies have shown that approximately 80% of China’s medical and healthcare resources are concentrated in urban areas, potentially limiting the accessibility of medical insurance for rural older adults populations and exacerbating health inequalities between urban and rural areas (56). As China’s aging population continues to grow, particularly among the oldest-old, health risks for older adults are increasing, driving a significant rise in the demand for healthcare services. In response, it is imperative for all regions to actively implement the directives of the Healthy China 2030 Plan and the 14th Five-Year Plan for Healthy Aging. These initiatives emphasize addressing the health challenges faced by key populations, such as older adults, improving older adults healthcare services, and promoting healthy aging. To achieve these goals, advancing the integration of urban and rural health insurance schemes should be a priority.

### 5.3 Strengths and limitations

This study utilized data from the latest nationally representative CHARLS survey released in 2020 to investigate cognitive trajectory changes in middle-aged and older adults individuals with dual sensory impairment. Based on the “Social Determinants of Health” model, it comprehensively and multidimensionally explored the predictive factors of cognitive levels in this population and proposed multidimensional strategies to improve their cognitive function.

However, this study has certain limitations. First, while it contributes to identifying risk factors for cognitive function among Chinese individuals aged 45 and above with sensory impairments, most of the risk factors are self-reported. Recall bias is unavoidable, as participants might overestimate or underestimate their visual and auditory abilities. Future research should incorporate objective assessments of hearing and vision, such as pure-tone audiometry, distance and near vision tests, and contrast sensitivity measurements, to validate and expand upon the findings of this study. Secondly, this study only assessed hearing and vision conditions at baseline, making it difficult to understand the dynamic changes in hearing, vision, and cognitive levels over time. Future studies should explore the bidirectional dynamic trajectories of hearing, vision, and cognitive impairments using longitudinal study designs with multi-trajectory modeling methods to evaluate how these conditions progress simultaneously. Finally, although this study demonstrates an association between hearing and vision impairments and cognitive levels, reverse causality may exist. To reduce this possibility, participants with very low baseline cognitive scores were excluded. However, this does not entirely eliminate the potential for confounding factors and reverse causality. Future studies should include larger populations with dual sensory impairments and conduct multicenter studies with large samples to verify these findings. Despite these limitations, the CHARLS database used in this study covers nearly all regions of China and is nationally representative. The predictive factors identified for the impact of dual sensory impairment on cognitive trajectories provide a reliable basis for further research.

## 6 Conclusion

In summary, this study found that older adults with dual sensory impairment aged 45 and above in China exhibit various cognitive development trajectories, indicating a heterogeneous process. Over the eight-year period, most of these older adults showed a decline in cognitive ability, with a significant proportion starting with already low baseline cognitive function. Factors such as nighttime sleep of less than 4 h, low social participation, alcohol consumption, and lack of health insurance are modifiable risk factors for cognitive decline in this population. Therefore, preventive strategies should be developed accordingly, particularly targeting vulnerable groups, including older adults rural residents and those with lower educational levels.

## Data Availability

The raw data supporting the conclusions of this article will be made available by the authors without undue reservation.
